# Primate phylogenomics: developing numerous nuclear non-coding, non-repetitive markers for ecological and phylogenetic applications and analysis of evolutionary rate variation

**DOI:** 10.1186/1471-2164-10-247

**Published:** 2009-05-26

**Authors:** Zuogang Peng, Navin Elango, Derek E Wildman, Soojin V Yi

**Affiliations:** 1 School of Biology, Institute of Bioscience and Bioengineering, Institute of Biosystems, Georgia Institute of Technology, Atlanta, GA 30332, USA; 2 Center for Molecular Medicine and Genetics, Wayne State University School of Medicine, Detroit, MI 48201, USA

## Abstract

**Background:**

Genetic analyses are often limited by the availability of appropriate molecular markers. Markers from neutrally evolving genomic regions may be particularly useful for inferring evolutionary histories because they escape the constraints of natural selection. For the majority of taxa however, obtaining such markers is challenging. Advances in genomics have the potential to alleviate the shortage of neutral markers. Here we present a method to develop numerous markers from putatively neutral regions of primate genomes.

**Results:**

We began with the available whole genome sequences of human, chimpanzee and macaque. Using computational methods, we identified a total of 280 potential amplicons from putatively neutral, non-coding, non-repetitive regions of these genomes. Subsequently we amplified, using experimental methods, many of these amplicons from diverse primate taxa, including a ring-tailed lemur, which is distantly related to the genomic resources. Using a subset of 10 markers, we demonstrate the utility of the developed markers in phylogenetic and evolutionary rate analyses. Particularly, we uncovered substantial evolutionary rate variation among lineages, some of which are previously not reported.

**Conclusion:**

We successfully developed numerous markers from putatively neutral regions of primate genomes using a strategy combining computational and experimental methods. Applying these markers to phylogenetic and evolutionary rate variation analyses exemplifies the utility of these markers. Diverse ecological and evolutionary analyses will benefit from these markers. Importantly, methods similar to those presented here can be applied to other taxa in the near future.

## Background

The accumulating body of draft genome assemblies from diverse animal species offers unprecedented opportunities for resolving the tree of life. A key component of empirical studies of molecular evolutionary phenomena is the analysis of molecular markers. To date, most molecular phylogenetic studies have relied on sequences from less than a few dozen genes. Mitochondrial DNA sequences have been the workhouse of phylogenetic and phylogeographic studies for the past two decades (e.g. [1,2]). DNA barcoding, a technique is increasingly used to identify species, is reliant on mtDNA [3]. While these methods have strengths, each carries some implicit limitations. First, because mtDNA markers are maternally inherited, the ability to infer evolutionary events from the perspective of both sexes is limited. In addition, the reduced effective population size of mtDNA compared to that of nuclear markers could confound population genetic inferences. Moreover, it is now well established that mtDNA sequences are often incorporated into nuclear genomes in diverse taxa, including humans and other primates [4,5].

Markers from single-copy nuclear DNA are free from the aforementioned problems. Often used single-copy nuclear DNA markers include conserved exons and genes. However, the effects of natural selection on these markers can result in homoplasy that has the potential to mislead phylogenetic analyses [6]. Similarly, genes that experienced positive selection in specific lineages (e.g., RNases evolution in leaf monkeys, [7]) may have inaccurate phylogenetic signals (i.e., they suffer long branch attraction due to increased number of nonsynonymous substitutions in specific lineages). Conversely, genes that have a history of strong purifying selection may harbor few phylogenetically informative sites, which make them unsuitable for population genetic studies or phylogenetic resolution in rapidly evolving taxa.

In addition to sequence based markers, events such as the insertion of transposable elements into ancient genomes provide excellent phylogenetic information [8]; yet these markers provides little information on rates of nucleotide substitution. Because of these limitations, neutrally evolving nuclear DNA sequence markers may provide the best source of data for phylogenetic inference and estimates of evolutionary rate variation.

Advances in genomics give molecular evolutionary studies an extraordinary opportunity to establish numerous nuclear, putatively neutral molecular markers. Genomes of many taxa, including those of primates, have a large amount of non-coding DNA, which can be used to infer genomic divergence and the influence of neutral mutation rate variation [9-11]. Therefore, we can obtain large numbers of putatively nuclear molecular markers from non-coding regions. Even though currently most taxa lack genome scale information, sequencing technologies are rapidly improving, and it will become progressively easier to obtain genome sequences. The challenges then are, to utilize genomic information to develop markers that can be used in a variety of ecological, phylogenetic, and evolutionary applications.

Here we present a method for developing and utilizing numerous non-coding, non-repetitive markers in primates. The availability of whole-genome sequences of primates combined with their well-resolved phylogenetic relationships makes them an excellent model system in which to devise computational and experimental tools to search for useful molecular markers. Moreover, such markers from primate genomes are potentially useful because they could be applied to the several outstanding phylogenetic problems in primates (for example, [12-15]). Such molecular markers also could serve as a resource for understanding the genetic history of primate populations, a topic of study of interest to molecular ecologists, primate biologists, and anthropologists.

We demonstrate the utility of these markers by applying them to phylogenetic and evolutionary analyses. We first constructed a sample data set of ten non-coding, non-repetitive markers from ten diverse primate taxa, including a strepsirrhine species rather distantly related to existing genomic resources. We reconstructed the correct species phylogeny with high confidence and uncovered significant evolutionary rate variation between lineages. Furthermore, we have uncovered new and statistically significant rate variation between some primate lineages. Thus, our markers may contribute understanding the patterns and causes of neutral evolutionary rate variation between lineages. We propose that the methods outlined here can be used in diverse taxa to address phylogenomic and population genetic questions.

## Results

### Amplification of potential markers

Our preliminary screen for orthologous non-repetitive and non-coding segments based on a three-way alignment the genomes of two hominid apes (human, *Homo sapiens*; and common chimpanzee, *Pan troglodytes*) and the rhesus macaque (*Macaca mulatta*, an Old World monkey) resulted in nearly 10,000 candidate amplicons. Subsequently, we designed 280 (212 from autosomes and 68 from the X chromosome) primer pairs in regions ranging from ~300 to ~1200 base pairs long, with an average length of ~600 bps.

We then used the polymerase chain reaction (PCR) to amplify these markers from the following primate species: gorilla (*Gorilla gorilla*, a nonhuman hominid), anubis baboon (*Papio anubis*, an Old World monkey), spider monkey (*Ateles geoffroyi*, a New World monkey) and tamarin (*Saguinus labiatus*, a New World monkey), and ring-tailed lemur (*Lemur catta*, a prosimian). Two hundred primer pairs (~70%) amplified a single band from *G. gorilla*, 170 primer pairs (~60%) amplified a single band for *P. anubis*, 116 (~41%) and 106 (~38%) primer pairs amplified a single band for *A. geoffroyi *and *S. labiatus *respectively. Furthermore, we amplified single bands in 18 markers (~6%), from a phylogenetically distant clade from the genome resources, *L. catta*. Sequences, locations in the human genome, and the applicable range of phylogeny of these markers (total 280 non-coding, non-repetitive markers) are listed the Additional file 1 [see Additional file 1].

We chose 10 markers (6 autosomal and 4 X-linked) for subsequent analyses (Table 1). These markers are likely to be orthologous, for the following reasons. First, these ten markers are unique genomic regions in the human genome (i.e. they are not included in segmentally duplicated regions, nor do they have closely related paralogous sequences in the genome, as determined by homology searches). Second, primers for all the ten markers produced single bands in PCR reactions in the primate species we tested. Third, when we used the experimentally amplified sequences as a query to search other genomes using BLAT or BLAST programs, all of these produced a single hit in the expected locations. Fourth, genetic divergences of these markers also fall within the expected ranges of neutrally evolving orthologous markers (see below). However, we caution that it is possible that these markers are not orthologous, given that most primate genomes are far from complete, and that unknown copy number variation may exist between and within species. Thus, whether the amplified markers of these regions from other primate genomes truly represent orthologous segments can only be determined by comparing finished, high-fold coverage, whole genome sequences from multiple individuals of the primate species in our study. The markers amplified and sequenced in this study are deposited in the GenBank (accession numbers GQ175181–GQ175229).

**Table 1:** The ten non-repetitive and non-coding (intron or intergenic regions) markers amplified and analyzed in this study.

*Marker*	*Corresponding human chromosome*	*ENCODE region*	*hg18 location*
A1	7	Yes	116019952–116020821
A2	10	No	130647230–130647739
A3	13	No	91672658–91673369
A4	15	No	84502360–84503129
A5	16	Yes	26155304–26155905
A6	21	Yes	34098146–34098878
X1	X	No	29000135–29000689
X2	X	No	31344540–31345210
X3	X	No	97879736–97880312
X4	X	No	141551659–141552259

We also retrieved these 10 regions from the publicly available genome sequences of the following five species: human (*H. sapiens*), common chimpanzee (*P. troglodytes*), orangutan (*Pongo pygmaeus abelii*), rhesus macaque (*M. mulatta*), and the common marmoset (*Callithrix jacchus*). All sequences, except for the X1 marker from the chimpanzee and the A5 marker from macaque, were of high quality (meaning, ≤ 2 ambiguous sites). The chimpanzee X1 and the macaque A5 marker had a substantial number of ambiguous sites, suggesting that the two particular markers may potentially include sequencing and/or assembly errors. Therefore, we experimentally amplified the two regions from the corresponding species and used them in further analyses.

Subsequent analyses were all based on these 10 markers. We used the GBlocks program [8] to remove poorly aligned sites. The alignment information and nucleotide content of the markers are shown in Table 2. A1 had the longest alignment with 778 aligned sites. The GC content of the 10 markers ranged from 30.5% in A3 to 54.3% in A2, with an average of 38.7% for the concatenated dataset. We tested whether base frequencies across taxa are homogenous using a chi-square test for each of the 10 markers and found no sign of heterogeneity among taxa (*P *> 0.1 for all markers, results not shown).

**Table 2:** Characteristics of the 10 non-repetitive and non-coding segments and for concatenated dataset.

				*Mean base frequencies (%)*				
					
*Locus*	*# aligned sites* *(# analyzed)*^1^	*# variable Sites*	*# PI sites*^2^	A	C	G	T	*Model*^3^
A1	806 (778)	271	96	32.1	23.5	19.5	25.0	GTR+G
A2	508 (508)	75	27	22.1	23.9	30.4	23.5	HKY+G
A3	485 (473)	151	63	31.4	15.6	14.9	38.2	GTR+G
A4	806 (673)	234	70	28.1	21.0	21.9	28.9	GTR
A5	619 (569)	205	83	32.9	18.0	16.3	32.8	GTR
A6	631 (569)	234	92	34.3	14.9	17.9	32.9	GTR+I
X1	508 (487)	145	63	28.5	18.0	18.6	34.9	GTR+G
X2	586 (565)	154	67	30.3	18.7	20.6	30.5	GTR+G
X3	466 (449)	125	43	33.5	17.0	17.1	32.4	GTR+G
X4	528 (522)	178	79	32.8	19.9	15.2	32.1	GTR+I
Concatenated	6535 (5592)	1791	684	30.6	19.3	19.4	30.7	GTR+G

^1 ^The number of analyzed sites are after removing poorly aligned sites using the Gblocks program [46].

^2 ^PI sites = parsimony informative sites

^3 ^Best fitting substitution models determined using the AIC (see text). (GTR = General time reversible model; HKY = Hasegawa-Kishono-Yano model; G = The shape parameter of a gamma distribution; I = The proportion of invariable sites in the alignment)

### Genetic divergence

To estimate the genomic divergence for these newly determined markers, we calculated the pairwise Kimura two-parameter distances [16] among the 10 primate species. A subset of pairwise divergences is shown in Table 3. All pairwise distances for the species compared here are shown in the Additional file 2 [see Additional file 2].

**Table 3:** Pairwise divergences between some primate species and groups. The Kimura two-parameter model [16] was used to estimate the divergence.

	***Kimura two-parameter Distance (×10^-2^)***										
	
***Marker***	H-C	H-O	H-MC	H-BA	H-MR	H-SM	H-TA	H-LM	HOM-OWM	HOM-NWM	OWM-NWM
A1	1.30	5.08	8.63	7.18	12.11	11.77	10.64	22.31	8.27	11.79	11.93
A2	0.59	2.00	2.32	2.11	4.91	4.06	5.12	9.31	2.35	4.78	4.33
A3	1.74	0.86	6.32	6.09	13.82	12.31	13.74	22.73	6.57	13.14	13.70
A4	1.96	1.97	6.82	6.14	10.80	10.23	10.39	24.50	6.83	10.94	9.60
A5	0.72	3.29	5.98	5.78	15.30	13.48	12.82	29.08	5.77	13.91	13.54
A6	2.16	4.04	8.28	8.69	17.39	15.18	15.00	34.78	7.36	14.94	16.50
X1	0.62	3.37	9.54	10.24	8.87	8.38	11.49	18.73	9.59	9.76	12.66
X2	2.34	3.64	7.25	6.66	11.24	9.97	10.55	17.71	6.40	10.38	10.97
X3	1.81	2.27	7.01	7.51	6.82	8.03	7.09	18.97	6.84	7.57	10.95
X4	2.16	5.02	9.33	8.89	10.44	11.91	12.55	21.86	8.39	11.83	14.28
Concatenated	1.53	3.24	7.29	6.88	11.22	10.60	10.81	22.07	6.92	10.90	11.80

Note. C = chimpanzee, H = human, G = gorilla, O = orangutan, BA = baboon, MC = macaque, MR = marmoset, SM = spider monkey, TA = tamarin, LM = lemur, HOM = hominoids, NWM = New World monkey, OWM = Old World monkey.

There is a substantial variation of evolutionary distances among markers. For example, the distance between human and chimpanzee ranges from 0.59% in A2 to 2.34% in X2. Such regional heterogeneity has been observed before and could be caused by several different factors, such as the difference in the proportion of sites susceptible to mutations caused by DNA methylation [17,18]. Nevertheless, this variation is within the range of 0%–2.91% observed in previous studies using non-repetitive and non-coding regions [9,19]. The mean human-chimpanzee divergence from these 10 segments is 1.53% ± 0.16%, which is close to previous estimates (1.24% ± 0.07% [9], and 1.19% ± 0.02% [19]). Thus, non-coding, non-repetitive markers developed in this study exhibit genetic divergences similar to those obtained from larger scale analyses.

We report that the divergence between baboon and macaque is 1.29% ± 0.16% for the concatenated dataset (range between 0.39% – 1.98%). This value is similar to an earlier estimate (1.24% in [17]). Within the New World monkeys, marmoset and tamarin are more closely related to each other (average distance: 4.02% ± 0.27%) than either is to the spider monkey (Table 3). This observation is concordant with the generally accepted understanding of platyrrhine phylogeny [14].

We also estimated genetic distances between different primate groups, defined as the mean of all pairwise distances between two groups in a inter-group comparison [20]. The average divergences between apes and Old World monkeys, apes and New World monkeys, and Old World monkeys and New World monkeys are 6.92% ± 0.34%, 10.90% ± 0.39%, and 11.80% ± 0.47%, respectively. Notably, genetic divergences between the spider monkey and the other two New World monkeys (spider monkey versus marmoset [5.85% ± 0.35%]; spider monkey versus tamarin [5.35% ± 0.32%]) are comparable to the divergence between apes and Old World monkeys (Table 3).

Our data also provide a chance to examine the genetic divergence between ring-tailed lemur and other primate groups based upon nuclear, non-coding, non-repetitive loci. Hominid apes, Old World monkeys and New World monkeys are on average 22.29% ± 0.75%, 23.66% ± 0.89% and 23.45% ± 0.74% divergent from the ring-tailed lemur based on our data. Previous studies suggested approximately 20–21% divergence between human and the ring-tailed lemur [12,21]. Our estimate of the genetic distance between hominids and the ring-tailed lemur is similar to this value, and these findings also show that Old World monkeys and New World monkeys have diverged slightly more from lemur than have the apes.

### Phylogenetic analysis

We performed phylogenetic analyses using the new markers generated in this study. We used Maximum Parsimony (MP), Neighbor Joining (NJ), and Bayesian Inference (BI) methods to construct phylogenetic trees for each of the 10 markers. The best-fitting substitution model for each marker for Bayesian Inference (BI) was determined by the Akaike information criterion (AIC) as implemented in ModelTest3.7 [22] and MrModelTest2.3 [23] (listed in Table 2). We also performed phylogenetic analyses using the three methods and a maximum likelihood (ML) method of the concatenated data set.

**Figure 1:**
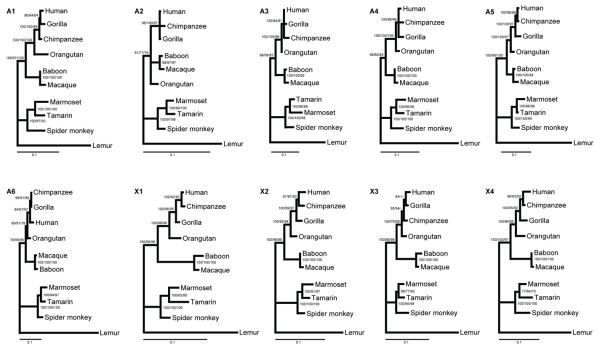
**Phylogenetic trees for each marker**. The numbers at each node were the support values based on Bayesian inference (BI)/maximum parsimony (MP)/neighbor-joining (NJ) analyses, respectively. The support values <50% were indicated using short dash "-" as shown in the figure.

**Figure 2:**
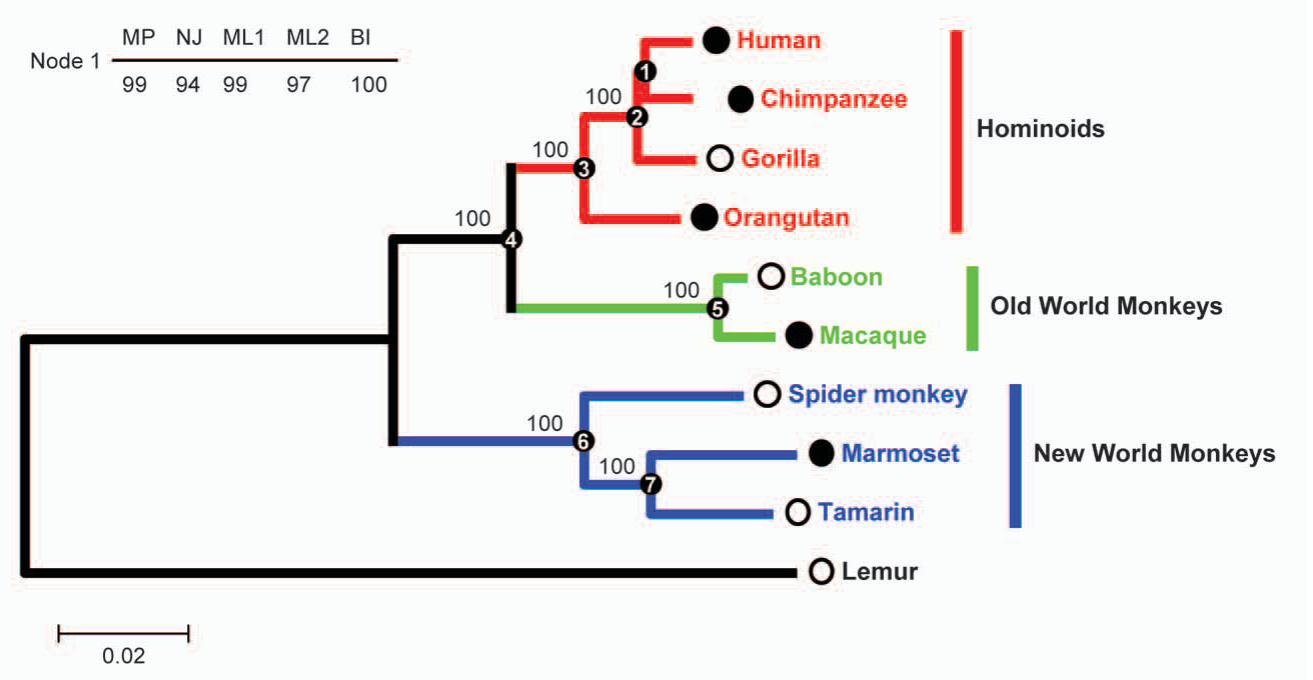
**Phylogeny of the ten primates**. The tree is derived from the analyses of the concatenated dataset (5592 bp) based on maximum parsimony (MP), neighbor-joining (NJ), maximum likelihood (using Garli [ML1] and PHYML [ML2]), and Bayesian inference (BI). All the nodes (except node 1, which indicated otherwise) received 100% bootstrap proportion and 1.0 Bayesian posterior probability support. Branch lengths were optimized using NJ based on Kimura 2-parameter distance model. The solid circle before the taxon name means the data for this taxon were retrieved from UCSC genome browser, while open circle means the data were sequenced in present study. As for the three ENCODE region markers (A1, A5, and A6), these segments for baboon were retrieved from NCBI.

Overall, none of these methods strongly supported conflicting topologies for any single marker. However, individual-marker analyses were less well resolved than the tree based on the concatenated data (compare Figure 1 with Figure 2). Figure 2 represents the total evidence tree obtained from MP, NJ, ML, and BI of the concatenated dataset. All the methods recovered identical topology with 100% bootstrap value or 1.0 Bayesian posterior probability support for all clades, except for node 1 (Figure 2). This tree agrees with the current understanding of the primate species tree in most aspect.

The behavior of combining characters from different partitions was evaluated by examining the relative contribution, or utility, of data partitions to resolving relationships of the combined data set. Specifically, partitioned Bremer support (PBS) was calculated using TreeRot3 [24] using the method of Baker and DeSalle [25] to measure the relative contribution of each marker to node support. The larger the partitioned Bremer support is for a given partition at a particular node, the greater the relative contribution of that partition to the support of that node. The sum of all partition lengths for any given node will always equal to the decay index for that node on the total evidence tree [25].

The results of the PBS analysis are presented in Table 4. The PBS values for 7 nodes across all the 10 markers and summed PBS scores for each marker are presented to evaluate the contribution of a given marker to the overall support of the simultaneous analysis tree. In order of total level of support, marker A5 shows the highest degree of support (56) for the simultaneous analysis tree, while A2 shows the lowest degree of support (16.5). For comparison, we also summarized the relative contribution of the support for the total autosomal markers (A1-A6) and X chromosomal makers (X1-X4), the concatenated dataset from the six autosomal markers shows a larger contribution (258) to the whole tree than that from the four X chromosomal markers (175) (Table 4).

**Table 4:** Partitioned Bremer support (PBS) for the simultaneous analysis parsimony tree.

*Marker*	*Node 1*	*Node 2*	*Node 3*	*Node 4*	*Node 5*	*Node 6*	*Node 7*	*Total*
A1	0	7.5	11.5	7	22	6	12	66
A2	0	5	0.5	3	3	5	4	20.5
A3	0	2.5	6	6	10	13	4	41.5
A4	0	3	12	3	12	11	6	47
A5	3	7	6	15	9	17	5	62
A6	-2	6	2	11	15	21	4	57
X1	3	4	5.5	6	17	7	2	44.5
X2	2	4	4.5	7	15	15	4	51.5
X3	0	1	2.5	6	15	5	1	30.5
X4	2	3	5.5	11	23	11	1	56.5
Autosomes (A1-A6)	1	31	38	45	71	73	35	294
X chr. (X1-X4)	7	12	18	30	70	38	8	183
Concatenated	8	43	56	75	141	111	43	477

Note. All marker rows show the PBS for each marker for the corresponding nodes shown in Figure 2.

### Relative rate variation in primates

To determine whether the rates of nucleotide substitutions for these 10 non-coding fragments varied between lineages on the phylogenetic tree, we performed relative rate tests. For example, to examine rate difference between apes and Old World monkeys, we used marmoset (a New World monkey) or the ring-tailed lemur (a strepsirrhine) as the outgroup (Table 5).

**Table 5:** Relative rate test: Comparisons of evolutionary changes among several primate lineages.

***Lineage 1***	***Lineage 2***	***Outgroup***	***K1***	***K2***	***dK***	***sd_dK***	***P***	***Ratio***
OWM	HOM	Marmoset	12.20	11.14	1.06	0.36	0.003**	1.36
OWM	HOM	Lemur	23.48	22.20	1.28	0.42	0.002**	1.48
Macaque	Baboon	Marmoset	12.48	12.08	0.40	0.18	0.029*	1.93
Macaque	Baboon	Lemur	23.86	23.42	0.44	0.21	0.039*	1.99
Baboon	HOM	Marmoset	12.00	11.14	0.86	0.37	0.019*	1.28
Macaque	HOM	Marmoset	12.42	11.15	1.26	0.38	0.000***	1.43
Marmoset	Spider monkey	Lemur	23.83	22.77	1.06	0.44	0.015*	1.48
NWM	HOM	Lemur	23.34	22.06	1.28	0.50	0.011*	1.24

Notes. 1. K1, number of substitutions per site that lineage 1 accumulated starting from the split with lineage 2; dK, the difference between K1 and K2 numbers of substitutions; sd_dK, standard deviation for dK; P, significance value for differences in substitution rates between lineage 1 and lineage 2; Ratio, the ratios of the branch length leading to the lineage 1 to that leading to the lineage 2 since the divergence of the two lineages.

2. HOM = hominoids, OWM = Old World monkey, NWM = New World monkey.

3. *significant at 0.05 level; **significant at 0.01 level; ***significant at 0.001 level.

Pairs of lineages that exhibit significant rate differences are presented in Table 5. We observe that the Old World monkey lineage (as a group) is evolving significantly faster than the ape lineage. The lengths of Old World monkey-specific branch are 30–50% longer than the ape-specific branch since their divergence, depending upon the choice of the outgroup (marmoset and lemur, respectively). This finding is in accord with the repeatedly observed 'hominoid/hominid-rate slowdown' phenomenon [17,19,26-29].

Interestingly, we found that the macaque lineage is evolving significantly faster than the baboon lineage since the divergence of the two Old World monkey lineages. In the ten markers studied here (Table 5) the macaque lineage has accumulated almost twice as many nucleotide substitutions as has the baboon lineage. Indeed, analyses of ENCODE data have shown that the branch length leading to macaque is significantly longer than that leading to baboon [30]. Another recent study using over 8 million base pairs of aligned genomic sequences among several Old World monkeys also indicated significant rate difference between rhesus macaque and baboon [31]. Overall, New World monkeys evolve at a faster rate than the hominids (Table 5). We also discovered significant rate variation among New World monkeys. In particular, marmoset has a significantly faster substitution rate (p = 0.015) than the spider monkey (Table 5).

These findings indicate that significant rate variation between lineages is a common feature of primate genome evolution. Based upon the known differences in life history traits [see Additional file 3], the observed rate differences are in general in agreement with the idea that species with larger body size, and likely longer generation time, tend to have slower molecular clocks. Baboons are larger and have longer lifespans than macaques, and spider monkey are larger and have longer lifespans than marmosets.

Taken together, these findings not only suggest a widespread influence of life history traits on molecular evolution of primates, but also provide a practical explanation on the fact that the degree of rate difference between specific primate groups can differ depending upon the actual lineages compared. For example, the hominid rate slowdown is more pronounced when rates are compared between human and macaque than between human and baboon (Table 5), which is in accord with the rate difference between macaque and baboon.

## Discussion

Genomics is arguably one of the fastest evolving branches of modern science. Emerging new sequencing technologies enable analyses of large number of individuals from a species or to interrogate genetic diversity of a complex biological community. Yet, aside from the fields of microbial genomics and human population genomics, for most taxa on earth only a sparse amount of genomic resources are available.

Developing genome-scale markers from the majority of the diversity of life will have high payoff, facilitating ecological and evolutionary applications. Here we have illustrated that we can develop markers from specific genomic regions (such as non-coding, non-repetitive regions) utilizing genomic resources that are moderately divergent from target species. For example, studies estimate that the human/chimpanzee/macaque and the ring-tailed lemur has shared the last common ancestor up to 80 million years ago [32]. Here we have generated markers from potentially neutral genomic regions in ring-tailed lemurs using primers based upon human-chimpanzee-macaque genome alignments.

As far as we are aware, data from non-coding regions of strepsirrhines are rare, and markers developed in this study have a great potential to be used in evolutionary studies of this group. Importantly, we demonstrated that we could potentially achieve genome scale marker generation by sampling genomes across the tree of life with moderate divergence times.

We chose Primates as the test group of organisms for this study because their phylogenetic relationships are well characterized [33]. We felt that if we could replicate the known primate phylogeny using the markers we designed then we would have proof of principle that this type of marker was useful for phylogenetic analysis. Toward that end this study was successful. Using a subset of new markers, we have performed phylogenetic analyses. The resulting phylogenies from single markers were generally in accord with the accepted phylogenetic relationship among different primate species. In particular, no marker supported incorrect phylogeny with statistical significance. The only node that required several markers to be resolved was the relationship among human, chimpanzee, and gorilla, a notorious phylogenetic example that had previously been shown to require a large amount of data to be resolved [34,35]. Given that there are several outstanding problems remaining in the field of primate phylogeny (e.g., [13-15,36]), putatively neutral markers such as developed in this study potentially will be useful toward resolving these issues.

Previous work focusing on identifying conserved, ultraconserved, or lineage specific elements for their potential functionality (e.g. [37,38]) have recognized the usefulness of utilizing regions located distantly from annotate genes as putatively 'neutral' standards to generate statistical 'background' for their analysis. Our approach, while seeking to identify regions of the genome that are not under functional constraint instead, have adopted such underlying logic and applied it to species whose genomic sequences are not yet available. It should be cautioned however that computational logics identifying putatively 'neutral' markers do not guarantee true neutrality: it remains as a prime challenge in modern genomics to experimentally establish neutrality or functionality of non-coding regions.

It has recently been demonstrated that protein coding regions are subject to frequent and widespread parallel evolution [6]; therefore we attempted to choose regions that are less likely to be subject to homoplastic effects that could result in misleading phylogenies. That our results support the well-known topology of Primates (e.g. [33]) is encouraging, because our understanding of primate phylogeny has taken over a century to get to the point is at today. There are still phylogenetic controversies (i.e. the hominoid trichotomy, the relationships among neotropical platyrrhines, and the phylogenetic placement of tarsiers) that still resist resolution besides decades of work.

Using the newly developed markers, we also observed substantial evolutionary rate variation among different primate lineages. We not only confirm the phenomenon of hominid rate slowdown [17,19,26,29], but also uncovered several other intriguing patterns. In particular, we observe that since the divergence of rhesus macaque and anubis baboon (estimated to be approximately 6–8 millions of years ago, [28,29]), the macaque lineage has accumulated almost twice as many mutations as the baboon lineage. Substantial rate variation between Old World monkey lineages could have contributed to earlier controversy over the degree of hominid rate slowdown. When the macaque lineage is used to compare evolutionary rates of hominids and Old World monkeys, the degree of rate slowdown is much stronger (Table 5). We also observed a strong rate difference between marmoset and spider monkey, and to a lesser degree between tamarin and spider monkey. The marmoset lineage has accumulated almost 50% more mutations than the spider monkey lineage since the two lineages have split (Table 5). Thus, evolutionary rate variation is a common phenomenon in putatively neutral genomic regions [18].

The relative rate test shows that mutations have accumulated in the Old World monkey and New World monkey lineage at a rate significantly higher than in the hominids. We found no significant evolutionary rate difference between Old World monkeys and New World monkeys, in contrast to an earlier finding [28]. However, we should be careful in concluding the patterns of rate variation between groups based upon data from a few lineages. First there is the issue of regional rate variation within a genome. Also, as we have witnessed above, rates can vary dramatically between closely related lineages (such as macaque and baboon). The earlier analysis on rate variation between marmoset and Old World monkeys [28] was based upon a single, albeit long (~59.8 kbps), orthologous region. The New World monkey species used in the previous analysis was marmoset, a fast evolving lineage. Thus the previous finding of significant rate variation between Old World monkeys and New World monkeys [28] may reflect the underlying molecular clock specific to that genomic regions and the sets of species. In this respect expanding the usage of non-coding, non-repetitive markers from many different genomic regions as developed here to other primate lineages will be highly useful to reconcile these conflicting results and to elucidate the patterns and causes of genomic neutral molecular clocks in primates.

The markers developed in this study [see Additional file 1] should thus be of great use for ecological and evolutionary applications in primates. In particular a subset of markers from closely related species can be used as 'local' markers to elucidate recent evolutionary events, while a few markers that can be amplified throughout the evolution of primates (such as the 10 markers analyzed here) could be used as 'global' markers to analyze underlying trends in primate evolution.

## Conclusion

Molecular markers from nuclear, putatively neutrally evolving genomic regions are extremely useful in ecological and evolutionary applications, yet hard to obtain from the majority of taxa. Here we describe a method for developing numerous nuclear markers from putatively neutral regions of primate genomes. First we demonstrate that by combining computational and experimental methods, we can generate a large number of putatively neutral markers from diverse primate genomes. Our PCR analyses show that we can amplify numerous markers from several well-separated primate lineages. We chose a subset of ten markers among the newly developed markers and amplified them from five primate species. Notably, we were able to amplify and sequence a subset of these markers from a ring-tailed lemur, representing a lineage that diverged from the lineage leading species from which the primers were designed 80 million years ago. We show that these ten markers can reconstruct the phylogenetic relationships among the ten primate species with high confidence, and useful in analyses of evolutionary rate variation between lineages. In particular we uncovered substantial rate variation among lineages, both within and between different primate families. Thus, these markers can provide a snapshot of genomic divergences and are likely to be highly useful in diverse applications.

## Methods

### Genome-scale mining of potential non-repetitive, non-coding amplicons

Whole genome assemblies of human (*H. sapiens*), common chimpanzee (*P. troglodytes*) and rhesus macaque (*M. mulatta*) (*hg18*, *panTro2*, *rheMac2*, respectively) were retrieved from the UCSC genome browser [39]. The human-chimpanzee-macaque alignments were obtained using blastz program [40]. Using the 'Ensembl' and 'Known' gene annotations provided by the UCSC genome browser, we identified non-coding regions, which include intergenic regions (defined as regions that are at least 1500 base pairs [bps] away from any known or ENSEMBL gene) and introns. We removed first introns and short (less than 250 bps) introns from further analyses, since these may be under selective constraint to preserve regulatory signals. We then extracted intergenic and intronic amplicons of length between 500 to 1500 bps, flanked by highly conserved 30–50 bp regions to be used as primer sites. Amplicons within 150 bps from an intronic start site or end site were also removed. We further filtered amplicons so that at least 80% of the sites are aligned between human-chimp-macaque and at least 85% of the aligned sites are from non-repetitive regions.

We then selected candidate primers, by either directly using the highly conserved flanking regions or automatically designing primers based upon the potential amplicons sequences, using the software fastPCR [41]. After designing primer pairs, we used either the BLAT [42] or BLAST [43] programs to map the primer pairs and the potential amplicons back to the human and macaque genome, to select only those that have a single hit in both human and macaque genome. This step removes potential spurious PCR products due to the homology to the primer sequences. Furthermore, we used BLAST program to map the single hit primers against the supercontig data of marmoset genome (downloaded from Washington University Genome Research Center). We chose primer pairs that satisfy the following criteria only: for each amplicon, the forward and the reverse primer should hit the same contig in marmoset genome, and the length of marmoset high scoring pair (HSP) should be at least 20 bps in length.

### Amplifying candidate amplicons from diverse primate genomes

To demonstrate the usefulness of the markers developed by our computational approach, we constructed a ten-species data set for a subset of markers. This data set is comprised of experimentally gathered data from five species combined with computationally extracted data from additional five species. First, five species across diverse group of primates were chosen to test newly developed non-repetitive and non-coding nuclear primer pairs. These are, gorilla (*G. gorilla*, a hominid), anubis baboon (*P. anubis*, an Old World monkey), black-handed spider monkey (*A. geoffroyi*, a New World monkey), white-lipped tamarin (*S. labiatus*, a New World monkey), and ring-tailed lemur (*L. catta*, a prosimian). DNA samples used in present study were purchased from Coriell (Camden, NJ) either as a primate phylogenetic panel (PRP00001) or individually (e.g., PR00036: anubis baboon).

To amplify the markers, approximately 5 ng of genomic DNA was used as the template for a 10 ul PCR mixture. We used a "touchdown" PCR program with an initial annealing temperature at 60°C and temperature subsequently decreased by 1°C every cycle until the specified annealing temperature 50°C is reached. Each reaction was performed using the cycles of 30 sec at 94°C, 30 sec at 60-50°C, and 60 sec at 72°C, with an initial step of 3 min at 94°C and a final step of 4 min at 72°C. Polymerase chain reaction products were purified using ExoSAP-IT (USB, Cleveland, USA) and then sequenced on both strands. The PCR primers were also used as sequencing primers. We performed gel purifications for the following three markers: A3 fragment from the baboon, A4 fragment from tamarin, and the A1 fragment from the lemur.

After sequencing, we used the BLAT program [42] to confirm that there is only one significant hit for each individual marker. These were combined with computationally extracted data from five other primate species. Specifically, homologous regions for the corresponding amplicons were retrieved from the UCSC genome browser from three hominids, including human (*H. sapiens*), chimpanzee (*P. troglodytes*; we amplified the X1 experimentally), orangutan (*P. pygmaeus abelii*), and one Old World Monkey, rhesus macaque (*M. mulatta*; we amplified the A5 marker experimentally), and a New World Monkey, common marmoset (*C. jacchus*).

### Sequence analysis

Initial sequence assembly was carried out using the Sequencher software [44]. Full alignments were produced with MAFFT using the L-INS-i MAFFT option [45]. All alignments were checked by eye, adjusted where necessary. Poorly aligned regions were removed using the Gblocks 0.91b program [46] with the setting of relaxed selection of blocks [47]. The final concatenated sequence alignment that was used for subsequent analyses contained 5592 aligned positions. Compositional stationarity was explored using the Chi-Square test in PAUP*4.0b10 [48].

Phylogenetic analyses were conducted by equally weighted maximum parsimony (MP) method, with gaps treated as missing data using PAUP, and by neighbor-joining (NJ) method, based on Kimura 2-parameter model using MEGA4.1Beta [20]. Maximum likelihood analyses were performed using both Garli0.96 [49] and PHYML3 [50] programs. The mixed-model analysis was implemented in MrBayes3.1.2 [51]. The best-fitting substitution models for the ML and Bayesian analyses were chosen by the Akaike information criterion (AIC) implemented in ModelTest3.7 [22] and MrModelTest2.3 [23]. Statistical support for the resulting phylogenies was assayed by conducting 1000 bootstrap pseudo-replicates for MP and NJ analyses as completed in PAUP and MEGA, and 100 bootstrap pseudo-replicates for ML analyses as completed in Garli and PHYML. Mixed-model Bayesian analysis also employed locus-specific models comparable to those chosen for ML analyses of individual genes. In MrBayes3.1.2, two independent sets of MCMC chains were run, each with three heated and one unheated chain for 2 × 10^6 ^generations. These were sampled every 100 generations. All analyses employed the default flat Dirichlet priors. The stationarity of each run was assessed by monitoring the convergence of the standard deviation of split frequencies and by graphing posterior probabilities of both runs against generations. The trees and parameter values from the pre-convergence "burn-in" phases of the runs were excluded.

The behavior of combining characters from different partitions was evaluated by examining the relative contribution, or utility, of data partitions to resolving relationships within the simultaneous analysis tree by calculating the Partitioned Bremer support (PBS) using TreeRot3 [24].

Significance of evolutionary rate variation among lineages were tested by relative rate test, using the program RRTree1.1 [52].

## Authors' contributions

DW, ZP and SY conceived the study and ZP, NE, and SY designed the particular experiment for the current study. NE performed computational mining and ZP generated markers by experimental and computational methods. ZP, NE, DW, and SY wrote the manuscript. DW and SY provided materials and reagents. All authors read and approved the final manuscript.

## Supplementary Material

Additional file 1 **Genomic non-coding, non-repetitive markers generated in present study**. Primer ID, corresponding human chromosome, and primer sequences are listed for the 280 markers. Click here for file

Additional file 2 **Pairwise Kimura two-parameter distances among the 10 primate species**. Pairwise Kimura two-parameter distances among the 10 primate species for each marker and for concatenated dataset are listed in the table. Click here for file

Additional file 3 **Life history traits among anthropoid primate species in this study**. Some important life history traits such as age of female sexual maturity, age at first birth, and lifespan are presented for the 9 ingroup species. Click here for file
