# Supplementary material for: Primate phylogenomics: developing numerous nuclear non-coding, non-repetitive markers for ecological and phylogenetic applications and analysis of evolutionary rate variation
Source: BMC Genomics. 2009 May 26;10:247. doi: 10.1186/1471-2164-10-247 (PMC2693144; doi:10.1186/1471-2164-10-247)
Supplement: Additional file 3 — Life history traits among anthropoid primate species in this study. Some important life history traits such as age of female sexual maturity, age at first birth, and lifespan are presented for the 9 ingroup species. [file 1471-2164-10-247-S3.doc]

Supplementary Table 3. Life history traits among anthropoid primate species in this study.

| Species | Male  Mass  (kg) | Female  Mass  (kg) | Infant  (months) | Female  Sexual  Maturity  (months) | Estrus  Cycle  (days) | Age at  First  Birth  (months) | Gestation  Length  (days) | Interbirth  Interval  (years) | Age at  Weaning  (days) | Lifespan  (years) |
| --- | --- | --- | --- | --- | --- | --- | --- | --- | --- | --- |
| Human | 68.23 | 55 | NA | 198 | 28 | 190-240 | 280 | 1-4 | 720 | 80-90 |
| Chimpanzee | 40-60 | 32-47 | 60 | 135 | 36 | 168-180 | 229 | 5 | 1440 | 53 |
| Gorilla | 169.5 | 71.5 | 36-48 | 78 | 32 | 102-132 | 256 | 4 | 1560 | 50 |
| Orangutan | 77.5 | 33-45 | 48 | 84 | 30 | 144-196 | 260 | 8 | 1260 | 59 |
| Baboon | 23.15 | 12.5 | 7 | 57.5 | 31-40 | 83 | 180 | 1.71 | 420 | 45 |
| Macaque | 9.36 | 7.09 | 12 | 42 | 29 | 54 | 164 | 1 | 316 | 29 |
| Spider  monkey | 7.78 | 7.29 | 24 | 72 | 26 | 60-90 | 229 | 2.9 | 822 | 27.3 |
| Marmoset | 0.32 | 0.329 | 2-3 | 22 | 13-15 | 20-24 | 148 | 0.52 | 91 | 11.7 |
| Tamarin | 0.49 | 0.53 | NA | NA | NA | NA | 145 | 0.82 | NA | NA |

Data sources from e.g. Lindenfors (2002) and references therein and Rowe (1996).

Lindenfors P. 2002. Sexually antagonistic selection on primate size. Journal of Evolutionary Biology 15: 595-607.

Rowe N. 1996. The pictorial guide to the living primates. Pogonias Press, East Hampton, NY.
